# “There Are Two Healing Processes in Cancer Care—There Is a Physical Healing and a Mental Adaptation Process”: A Pilot Study for Preparing Children and Adolescents with Osteosarcoma for Limb Amputation

**DOI:** 10.3390/cancers17172755

**Published:** 2025-08-24

**Authors:** Cynthia Fair, Bria Wurst, Lori Wiener

**Affiliations:** 1 Department of Public Health, Elon University, Elon, NC 27244, USA; bria.kaelyn@gmail.com; 2 National Cancer Institute, Center for Cancer Research, National Institutes of Health, Bethesda, MD 20892, USA; lori.wiener@nih.gov

**Keywords:** amputation, surgery, body image, mental health services, pre-surgery preparation

## Abstract

Background/Objectives: This study assessed how to best prepare pediatric and adolescent cancer patients for amputation and support them afterward. Methods: This pilot qualitative study explored pre- and post-amputation experiences from the perspectives of nine pediatric and adolescent survivors who underwent amputation. Hour-long audio-recorded semi-structured interviews were transcribed and analyzed using the Sort and Sift, Think and Shift qualitative approach. Results: Participants described the informational supports they received before surgery, including guidance on what to expect, contact with amputation-related organizations, and exposure to tangible tools, such as a physical model of a knee joint. Emotional support from fellow amputees and healthcare providers, particularly surgeons, was also found to be meaningful. Individuals also identified unmet needs and gaps in emotional care. These included clearer guidance on post-surgical adaptations (e.g., basic self-care and navigating physical limitations) and the need for information tailored to their learning styles. Many emphasized the importance of improved pain management resources, expanded access to mental health services for both them and their families, and support in adjusting to changes in body image and social relationships. Participants also shared advice for future patients, recommending strategies such as personalizing hospital rooms, connecting with other amputees through social media, and using art to process their experience and say goodbye to the lost limb. Conclusions: Interviews with nine cancer survivors provide guidance for improving holistic, patient-centered care throughout the amputation process. Informational and emotional support should be tailored to an individual’s learning style and specific needs, in addition to their age at the time of surgery.

## Simple Summary

Pediatric and adolescent patients with cancer who require limb amputation face significant physical and emotional challenges. While medical care has improved survival outcomes, far less is known about how to prepare young patients for the psychosocial impact of amputation and how to support their adjustment afterward. This study draws on the lived experiences of cancer survivors who underwent amputation during childhood or adolescence to identify key elements of informational, emotional, and social support needs. By listening to firsthand accounts, the study aimed to inform the development of more comprehensive, patient-centered care strategies. The findings highlight missed opportunities for improving mental health access, the benefit of tailoring information delivery to individual needs, and integrating peer and family support into clinical practice. These insights can guide clinicians, psychosocial professionals, and healthcare systems in enhancing the quality of care for future patients undergoing similar procedures.

## 1. Introduction

In the U.S., approximately 15,780 children aged 0–19 are diagnosed with cancer annually [1], with about 1 in 285 children receiving a diagnosis before their 20th birthday. Bone sarcomas, primarily osteosarcoma and Ewing sarcoma, represent 3% of pediatric cancers (roughly 400 cases/year in the U.S.) [1]. Although less common than other types, these cancers often require limb amputation in 9–57% of cases [2], along with radiation and chemotherapy, placing patients at a heightened risk for poor psychosocial outcomes [3]. Osteosarcoma is the most common malignancy of bone in children and young adolescents, most often occurring in the lower extremities, and accounts for approximately 60% of all malignant bone tumors diagnosed before young adulthood [4].

Research highlights the importance of pre-operative interventions in promoting coping and adjustment and reducing post-operative distress [5]. However, Weschenfelder et al. [6] identified a critical gap: no psychological interventions are tailored specifically for youth facing amputation, despite the potential traumatic nature of the surgery and aftermath [7]. While existing studies focus on post-operative quality of life, there is limited guidance on pre-operative support [6]. For localized bone sarcomas, the five-year survival rate is 76% [1], underscoring the need for effective psychosocial interventions to support long-term well-being.

This study aimed to identify essential components of pre- and post-operative support for pediatric and adolescent patients undergoing amputation, drawing on insights from individuals with lived experience. Qualitative methods are particularly well-suited for exploring understudied populations [8], as they provide rich descriptions and amplify the voices of individuals often excluded from research [9,10].

## 2. Materials and Methods

### 2.1. Participants

Nine English-speaking adolescents and young adults who underwent amputation due to cancer in their childhood or teen years participated in semi-structured, audio-recorded phone interviews. Interviews lasted approximately 60 min. Participants were recruited at the NIH Clinical Center, Dana-Farber Cancer Institute, Sydney Children’s Hospital, and Make it Better Agents. The study was open at three sites. Each site identified eligible participants (having undergone an amputation during their childhood or teen years). Due to the high relapse rate, each site had very few participants. There were no participants reported to us who, after being approached, declined to participate. Each site documented the patient’s participation and interview within their medical record or required systems.

### 2.2. Procedure

Clinicians trained in qualitative interviews conducted the interviews. Audio recordings were professionally transcribed. The NIH Office of Human Subjects Research determined the study to be exempt from full IRB review (OHSRP #5736). Participants did not receive any incentives or compensation for participation. Oral consent was obtained before recording each interview. See Supplemental S1 for oral consent procedures.

The interview was developed by psychosocial experts in the field of pediatric oncology. Interview questions (Supplemental Table S1) included how the surgery was explained to them, how they were emotionally and physically supported before and after amputation, and what advice they would give to children facing amputation and their parents. Sample questions included the following:

- “From what you know now, what do you feel would have been helpful for you to have been told before the time of amputation?”
- “Was there anything that the care team did or said that you remember helped you feel more prepared or comfortable? Please explain.”
- “Did you feel you were prepared emotionally for the amputation? After the amputation? Please explain.”

### 2.3. Analysis

Transcribed interviews were analyzed using the Sort and Sift, Think and Shift method, an iterative approach that encourages researchers to immerse themselves in the data to uncover its key dimensions and meanings, followed by deliberate reflection to interpret findings in relation to broader scholarly conversations [11]. In the initial phase of analysis, authors independently and then collaboratively read the transcripts in full to gain a deep understanding of the material and to identify emerging patterns related to support received and desired before and after the survey [12]. Summaries of each interview were created and organized into matrix form to facilitate cross-case comparison and identify trends and variations in participants’ experiences [13]. A preliminary codebook was developed based on the range and nuance of themes within each analytic domain. All transcripts were uploaded into Dedoose (Version 9.0.107, 2025) [14], and coding was carried out collaboratively through a consensus-driven process.

## 3. Results

### 3.1. Subsection

Nine pediatric and adolescent cancer patients with limb amputation completed in-depth interviews. Mean age at amputation was 15.2 years, and at the time of the interview, it was 25.1 years. Mean time between amputation and interview was 10.0 years. One participant identified as Asian, and the rest identified as White. Please see Table 1 for additional demographic information.

#### 3.1.1. Resources Offered: Informational and Emotional Supports in Place

Participants expressed gratitude for the informational and emotional support they received throughout the amputation process. Resources varied, with some receiving “what to expect” information, contacts from amputee organizations, and the opportunity to see a physical knee joint. One participant underscored the importance of timely communication, saying, “I definitely think that once the word is out, definitely someone should talk to the child. There are going to be questions; they are going to be filled with questions.”

Emotional support was offered through peer connections and medical care providers. Meeting other amputees was critical in preparing for treatment. “The idea of being able to connect with other amputees is so tremendously important. The internet affords you the ability to transcend physical separation and connect with other amputees.” One participant remarked that it was important that she spoke with an amputee who was also a woman, as she had specific questions about attire and navigating romantic relationships. Surgeons were seen as particularly helpful, as indicated by the following quote. “She was really good. I remember her sitting and like I was talking to her once. I mean all the doctors were. They really, really drummed into me the message that this would not stop you from doing anything you want to do.” See Table 2 for additional definitions and quotes.

#### 3.1.2. Resources Desired: Unmet Needs and Gaps in Emotional Support

Participants identified a range of needs that were not met, including strategies for self-care, adaptation to physical activity, and sensitivity to learning styles. Multiple interviewees mentioned the challenges of maintaining personal wellness after surgery, particularly bathing. “I think something that I didn’t know was that walk-in showers and tubs are a lot different with one leg.” Identifying strategies to continue participating in physical activities was an area of unmet need, as illustrated by this statement: “Then just learning how to walk again was hard. So, I think that time probably would have been the best to have a mentor. I didn’t see her after I had an amputation. Only before.”

A clear need for additional emotional resources before and after their amputation was emphasized. They specifically expressed the desire for more assistance in coping with pain and improved access to mental health providers for themselves and their families. Participants felt prepared for the surgical pain but not for phantom pains. For example, “I was really ready for it [the surgery] because I was in so much pain, but I did after have a mental breakdown because I was in so much pain. And the phantom pains were really bad for me.” The need for additional emotional support was expressed by multiple individuals. Participants wished for mental health services before and after surgery. One person explained, “But also I thought that that could have been better with like mentors, and psychologists, and other support of like mentally and emotionally because it was a big decision.” The desire for emotional support extended to parents and siblings. “Through my treatment or through my amputation, I think it would have been nice for them to have someone to talk to or like a group about it. I feel like the families are kind of forgotten in the whole process.”

Multiple participants emphasized the importance of adapting information to the learning styles and comfort level of each patient. For example, “I personally did not even want to know. Some people, they look up what an amputation surgery looks like because they want to know. I didn’t because I knew it was going to be not pretty. I liked being explained it better than looking at it”.

Further, participants sought support in building social connections, given their new physical restrictions, and coping with self-image challenges. Maintaining friendships through shared experiences was important to the youth. However, they struggled to find ways to connect with peers due to their new physical limitations, as previous pastimes had included hiking or other physically strenuous activities. Issues of self-image also emerged, as noted in the quotes below:

- “People pay more attention to me now that I have the leg, and I don’t know if I like that.”
- “It was a bit difficult I suppose like when going out in public. Because we were from a small town, so everyone knew what was going on. But, yeah, everyone saw that thing.”

See Table 3 for additional definitions and quotes.

#### 3.1.3. Reflections and Advice

Participants enjoyed the opportunity to reflect upon their journey and offer advice to other pediatric and adolescent cancer survivors with limb amputation. They suggested other cancer survivors “keep a journal throughout the process” and personalize their hospital room. Maintaining connections with other amputees through social media was also encouraged. Several noted the importance of finding creative ways to say “goodbye” to their limb. For example, “Then I also had people sign my leg, which I know is very popular. That was really helpful because it was just like saying goodbye to it. Instead of hating it and hating that the tumor was there, being thankful which was very, very helpful for me”.

See Table 4 for additional definitions and quotes.

Table 5 summarizes the helpful and unhelpful experiences reported by pediatric and adolescent cancer survivors who underwent limb amputation. Helpful experiences included access to clear information, supportive providers, opportunities for connection, and emotional validation, all of which facilitated adaptation and healing. In contrast, participants also described unmet needs, including insufficient self-care guidance, limited pain management strategies, difficulty maintaining social connections, and poor access to mental health resources. Together, these themes highlight both the strengths of current care approaches and the critical areas requiring further attention to optimize patient support.

## 4. Discussion

Little is known about how to best support pediatric and adolescent cancer patients facing limb amputation. Our findings suggest that informational resources, including access to cancer-related organizations, were readily available. Developmentally sensitive and honest communication is vital. Ongoing assessments of their level of interest and preferred learning style are necessary. Age is not always a reliable indicator of a child’s informational needs, as they can change over time. Assessment should take place before and after surgery, not only to evaluate a patient’s understanding of the treatment, but also to explore its emotional implications [15].

Participants found emotional support by communicating with other individuals who have undergone similar experiences. Peer modeling and connection helped foster a sense of normalcy and belonging [16]. Participants also received comfort from their medical care providers, who sought to underscore the message that their patients could lead rich and fulfilling lives. However, while positive messaging is essential, the data suggest that providers must avoid overly optimistic assurances that may downplay the challenges of recovery and adjustment. Realistic preparation, including discussions of adaptation and loss, is essential for psychological resilience [17].

Participants highlighted the urgent need for emotional support related to pain management and mental health access. While most anticipated surgical pain, they were unprepared for phantom limb pain, which has been described in 60–80% of amputees [18]. Phantom limb pain often requires a multimodal approach, including pharmacologic agents, such as gabapentin or opioids [19], and non-pharmacological approaches, such as acupuncture or mirror therapy, where patients perform symmetrical movements in front of a mirror to “trick” the brain into perceiving the movement in the missing limb [20]. Finally, psychological approaches to pain management, such as guided imagery or cognitive-behavioral interventions, have been recommended for pediatric cancer patients [21].

Mental health support was repeatedly cited as essential but underprovided. Youth expressed a need for access to psychologists before and after surgery to help process fear, grief, and identity changes. Family members also experienced distress, yet few had access to mental health support. This aligns with broader pediatric oncology literature showing that family-centered psychosocial interventions improve coping outcomes and reduce distress in parents and siblings [22]. Support groups, both online and in-person, can also foster a sense of community and reduce isolation. In recognition of these needs, the Standards for the Psychosocial Care of Children and their Families (the Standards), published in 2015, emphasized the importance of integrating mental healthcare throughout the cancer trajectory [23]. However, many cancer centers lack the staffing needed to translate the Standards into routine clinical practice [24].

Analyses revealed a tension between feeling visible and feeling vulnerable. This highlights the importance of providing anticipatory guidance on body image changes [25]. Pediatric studies show that children with visible differences often experience greater social anxiety and stigma, particularly during adolescence, when peer identity becomes paramount [26]. Interventions should include opportunities for open dialogue about body image, reassurance of worth beyond appearance, and guidance on how to discuss their bodies with peers.

Many participants reflected on the importance of personalization and closure. Small gestures, such as decorating their hospital rooms or journaling, offered agency during a time of medical powerlessness. Encouraging creative rituals, such as having peers sign a cast or taking a photo with the limb pre-surgery, helped promote closure and symbolic transition [27]. These practices can be incorporated into clinical preparation protocols.

Maintaining peer connections, particularly with other amputees, was another consistent theme. Participants noted that social media provided an accessible means to exchange advice, vent frustrations, and gain emotional validation. Structured peer mentorship programs may offer a formal avenue for these benefits [28]. Providers can also create or refer interested families to curated networks of age-matched peers who have undergone similar procedures. See Supplemental Table S2 for a list of nationally available resources.

### Limitations and Future Research

Several limitations need to be considered. The small sample size makes definitive statements about guidance challenging. Because interviews were conducted over the phone, nonverbal cues could not be captured. However, this qualitative study offers a springboard for future research with larger and more diverse samples.

Many participants discussed the importance of attaining good prosthetic function, but we did not capture detailed accounts from each individual regarding their prosthetic experiences. Challenges related to body changes and self-image also emerged as central concerns, underscoring the need for longitudinal research to better understand the physical and psychological adaptation to prosthetic use. Retrospective recall is another possible source of bias, though it offered participants the benefit of temporal distance from the immediate trauma of amputation, allowing for a more nuanced reflection on resources that supported their healing.

Selection bias is possible, as participants not only survived cancer but may also have been better adapted than those who did not participate. Another consideration is that participants underwent amputation in different calendar years, during which models of care may have varied. Prior exposure to intensive chemotherapy may have also influenced participants’ perspectives on amputation.

Our sample was relatively homogeneous in terms of racial and ethnic composition, compared to the broader epidemiologic distribution of pediatric and adolescent osteosarcoma, which disproportionately affects Black and Hispanic youth [29]. Efforts to include perspectives from a more diverse patient population are warranted, as existing literature demonstrates that marginalized groups often face discrimination in healthcare settings, which compromises access, quality of care, and outcomes [30,31]. Finally, perspectives of family members were not included. Retrospective and prospective research should seek to broaden the understanding of the needs of parents and siblings.

Despite these limitations, this study has important strengths. To our knowledge, it is among the first to elicit in-depth perspectives from pediatric and adolescent survivors of osteosarcoma who experienced limb amputation. By centering the voices of individuals with lived experience, the study provides valuable insights into both pre- and post-amputation needs. These findings offer a rare and meaningful foundation for developing patient-centered interventions that can improve preparation, rehabilitation, and long-term psychosocial support for this population.

## 5. Conclusions

Research suggests that amputations in childhood and adolescence can be particularly traumatic, yet no prior studies were found that examined what pre-surgery interventions youth find helpful. It was also not known whether individuals who had an amputation during their childhood or adolescence found being involved in the planning and decision-making process useful. This pilot study is the first available literature to describe preparation for amputation in youth with cancer. The findings underscore the need for holistic, developmentally tailored, and emotionally attuned care before and after amputation. Future intervention models should integrate psychoeducation, peer support, and mental health resources across the cancer journey.

## Figures and Tables

**Table 1:** Participant demographic information.

Participant #	Sex	Age at Surgery	Age at Interview	Type of Cancer	Type of Surgery
1	M	9	25	osteosarcoma	amputation
2	M	10	36	osteosarcoma	amputation
3	F	19	27	osteosarcoma	amputation
4	M	18	24	osteosarcoma	amputation
5	F	12	33	osteosarcoma	amputation
6	F	18	24	osteosarcoma	amputation
7	F	18	20	osteosarcoma	amputation
8	F	19	21	osteosarcoma	amputation
9	M	14	16	osteosarcoma	rotationplasty

**Table 2:** Resources offered: informational and emotional supports in place.

**Informational Resources**		
**Theme**	**Definition**	**Quotes**
What to expect	Participants appreciated information about how their bodies would look and feel after amputation.	“Yeah, I mean they showed me exactly where the tumor was—the bone growth—the cancer was. And then they showed me exactly where they needed to take—where they needed to amputate—how far up they would need to amputate my leg. And then, um, they also showed me what it would look like and what it would feel like and what my—what the post-surgery would be like if I had gotten it reconstructed.”
		“…with Dr. E [the surgeon] did give me pamphlets of what the stump would look like and how to clean it and all that.”
		“They told me about the drainage tube during surgery.”
Supportive organizations	Adolescents often mentioned organizations that offered emotional and physical support.	“I think having contact with foundations, like Amputee Coalition and MIB Agents…I think having a support network while you’re going through it and being able to have a place where you can ask questions and get opinions from the people who have already been through it is important.”
Physical materials	A few participants appreciated being shown a physical knee joint.	“Dr. M, she showed me, when she was explaining the limb salvage, she showed me the mechanical knee joint, the actual one that she was going to put inside me if I decide on that surgery.”
**Emotional Resources**		
**Theme**	**Definition**	**Quotes**
Connecting with others	This theme referred to the importance of connecting with other amputees and having role models with similar experiences for guidance and support.	“Being involved with Special Op is still … one of the biggest parts of my joy. It’s great to see when you’re there to see other people, and then you also have role models. R. was one of my role models. And to see him having life afterwards is a wonderful thing.”
		“There’s a guy named L., and he drives a white convertible, and he works for the government in DC and he’s very handsome, in his late 20s. And just this picture of this adult guy with one leg who had a job and was very handsome and had a white convertible, just that concept was like amazingly powerful.”
Support from medical providers	Participants appreciated the emotional support, particularly from their surgeons, during visits and hospital stays.	“I think just my surgeon was there for me. After my amputation, she gave me a gift and it just meant a lot because she was not just my surgeon, she was human and she saw me as a human, too.”
		“My family and I had talked about amputation. One [children’s hospital] was against the amputation, and I had a lot of pushbacks from them. So, I actually left and went to another hospital and had it done. But my decision to [move care] with the healthcare provider was not a good interaction. I walked out very angry and upset [with the first hospital]. But then, when I went to the other one, it was a totally different experience.”

**Table 3:** Resources desired: unmet needs and gaps in emotional support.

**Unmet Needs**		
**Theme**	**Definition**	**Quotes**
Self-care	Basic personal wellness needs, specifically showering, were frequently mentioned as a desired resource.	“I mean we had to modify the bathroom, which we did beforehand. So that was one thing that they had suggested to mom and dad. But I don’t know what they really talked about or said.” “PT started having me learn how to do stairs in adaptive ways. I think there could be more physical therapy towards household ways of doing things. We didn’t talk about showering but maybe how to transfer from a chair into a shower.”
Physical activity	Participants expressed a desire to learn how to modify previously enjoyed physical activities.	“I’m a really active person and having to stay still and lay in bed all day for months at a time was just unbelievable for me.” “The dancing is a big thing to me, ……over sort of college, I started to get more confident, and now I go out dancing with my friends over the weekend.”
Learning style	Participants wished that providers adapted information to different learning styles of patients and families.	“I’m the kind of person who likes people telling me more than reading stuff. But my parents found it very helpful.” “I’m a visual learner. I watched videos after I spoke with the doctors.”
**Desired Emotional Resources**		
**Theme**	**Definition**	**Quotes**
Coping with pain	Participants expressed the need for more information about managing physical and phantom limb pain.	“I guess the nerve pain was really bad. I still deal with it to this day. I guess that could’ve been explained better just because it’s such a weird sensation. I didn’t expect to feel so terribly from that.” “I think one of the biggest shocks was the phantom pain. I don’t think that term gives it enough credit because it’s not fake pain. It’s very much real and I think I didn’t have the coping skills that I wish that I did have for that.”
Access to mental health providers for patient and family -Patients -Families	Participants emphasized the need for mental health professionals to provide dedicated emotional support, noting that while medical providers were supportive, they lacked specialized training in addressing the psychological impact of limb amputation. Many also wished their families had received more emotional support during the process.	“I think definitely the biggest thing would be a mentor and social support. I think it would be good if a prosthetic company came before and actually had you come and look. I feel like the surgeon though was just doing the surgery, but there was no psychologist. It is such a big emotional change.” “There are two healing processes in cancer care. There’s a physical healing and a mental adaptation process and I wished I had more emotional help.” “There was no counseling. There was nothing at all.” “I feel like if there was a team of people, that would help out a lot to get them prepared and explain to them what’s going to happen. Because the unknown is a lot scarier than knowing what’s going to happen.” “They have seen me having two legs all my life. So, seeing me without one of them would be like a very hard thing on them basically.” “It was really, really, hard for him (7 years old brother) I think probably just because I was getting all this attention, and he wasn’t and partly because he didn’t quite understand what was really going on and he thought I might die, and we were like best friends.”
Body change support -Social connection -Self-image	Participants highlighted social connection and self-image as key post-amputation challenges, citing isolation from reduced peer activities and emotional difficulty adapting to bodily changes and others’ perceptions.	“My friend, she was going to Aruba. She was like ‘I just want you to know that you’re completely invited. But I’ve scoped it out. It’s not very wheelchair accessible.’” “My friends right now are planning a hike to the Grand Canyon. That really hurt me because they didn’t even invite me obviously. There’s just stuff that I can’t do which I’m still not really knowing how to deal with yet because every amputee that I talk to deals with the same thing.” “Because I have the prosthetic they are looking at me and like trying to figure out what I am doing.” “The limb doesn’t stop changing for the first year to two years. I was really expecting it to stop in six months. And my limb is still changing for almost two years now. I think, just to know that that’s normal and that it is a long process, but it will eventually even out, I think would have been helpful.”

**Table 4:** Reflections and advice.

Theme	Definition	Quotes
Personalization	Participants found personalization helpful as a coping strategy, engaging in creative activities, customizing their environments, and connecting with others through support groups or social media.	“Every single hospital stay I have different decorations in my room for something to look forward to. Because you don’t want the room to look so blah. When you’re going in for amputation, you want a nice and bright comforter and a couple of decorations here and there.”
		“I know there’s a couple [amputation groups] on Facebook. Some people have osteosarcoma, some people have been in accidents, but they’re all dealing with amputation. Everyone always has all these different facts and all these useful links. It’s just very useful.”
Closure	Several participants discussed the importance of saying goodbye to their limb through creative activities.	“I think the most important thing to start off with is how can you get closure for the process. The way that I did closure was saying goodbye to my leg, and being thankful, and doing all those things. Like parasailing and going up in a helicopter. Just really having fun before things got serious basically. Putting paint all over my foot and doing this really cute picture so I’ll always have my footprint. Making little jokes. Like, hey, you can get 50 percent off of pedicures now. Little jokes like that and finding the right person to talk to.”
		“I painted my foot with different watercolors and then I put it on a piece of paper. I have that in my room. It’s really powerful to me to just see. It’s not just like a footprint on the wall. It’s like watercolor and it’s beautiful. That was really helpful. It was kind of like a fun activity to do as well.”

**Table 5:** Summary of helpful and unhelpful experiences.

Helpful Experiences	Unhelpful Experiences
Clear and timely informational resources about care (pre-surgery)	Educational materials not adapted to age, learning style, or developmental stage (pre-surgery)
Opportunities for personalizing the care process and achieving closure (e.g., memory-making, rituals) (pre-surgery)	Poor access to mental health services for patients and families (pre- and post-surgery)
Supportive, attentive medical providers (pre- and post-surgery)	Lack of practical information on self-care and recovery (post-surgery).
Connection with peers and others with similar experiences (pre- and post-surgery)	Limited strategies offered to manage acute and chronic pain (post-surgery)
Access to supportive counseling and emotional care (pre- and post-surgery)	Difficulty regaining physical activity and mobility (post-surgery)
Encouragement and validation from family and providers (pre- and post-surgery)	Challenges maintaining social connections and coping with body image/self-esteem concerns (post-surgery)

## Data Availability

Data available upon request.

## Supplementary Materials

The following supporting information can be downloaded at: https://www.mdpi.com/article/10.3390/cancers17172755/s1. Supplmentary S1: IRB notification and Oral Consent Procedures; Table S1: Interview Protocol; Table S2: Current Resources for Children and Adolescents with Osteosarcoma.
