# Supplementary material for: “There Are Two Healing Processes in Cancer Care—There Is a Physical Healing and a Mental Adaptation Process”: A Pilot Study for Preparing Children and Adolescents with Osteosarcoma for Limb Amputation
Source: Cancers (Basel). 2025 Aug 24;17(17):2755. doi: 10.3390/cancers17172755 (PMC12427263; doi:10.3390/cancers17172755)
Supplement: Supplementary file 1 [file cancers-17-02755-s001.zip › cancers-3767895-supplementary.pdf]

cancers MDPI

Supplemental S1: IRB notification and Oral Consent Procedures

IRB Notification

Attached is the OHSRP determination of Excluded from IRB Review per 45 CFR 46 for your project, Pediatric Amputation: The Experience of Pediatric Amputee and Current Clinical Practice. You may proceed with the project.

Please retain this documentation as you would other research records. Amendments and or changes to the research must be submitted to OHSRP for review as changes may affect the determination.

Please refer to OHSRP #5736 for future amendments to this activity.

Exempt research under 45 CFR 46

 https://doi.org/10.3390/cancers17172755

Oral Consent Script

Thank you so much for participating in this interview. Before we begin, I want to review why we invited you and formally obtain your consent to participate. As we go along, please feel free to stop me if you have any questions.

The purpose of this study is to learn as much as we can about how to best prepare children and adolescents with cancer for an amputation. Having gone through an amputation when you were younger, makes you an expert! We are so appreciative of your time and willingness to help others.

I will be asking you a number of questions. Please let me know if any of the questions are confusing so I can clarify what we are asking. Please also let me know if any of the questions are emotionally difficult. We can stop the interview at any time.

We hope to record this interview so that we can pay full attention to what you are saying and not have to spend time taking notes. All information you provide is confidential. No one outside of the immediate research team will have access to this recording.

The recording and transcript will be destroyed when the study data analysis is completed. We may share your data with other researchers or persons who help youth prepare for amputation. When we share your data, we will protect your identity. We remove your name and any information that can identify you.

Are you comfortable with this? Would you still like to proceed? Do you have any questions for me before we begin?

Supplemental Table S1 Interview Protocol

Screening Tool

Participant ID: _________ Age:_______ Gender: _____ Date

Instructions: Read each statement and decide if that statement describes how you feel. Check "yes" to indicate that the statement is true for you and "no" to indicate one that is not true for you.

1. I feel comfortable talking about the preparation that took place prior to my amputation.
Yes_______ No _______

2. Talking about my amputation still upsets me very much.
Yes_______ No _______

3. I feel comfortable discussing what I remember about the preparation that took place prior to my surgery.
Yes_______ No _______

4. I feel comfortable discussing what I would recommend health care providers do to help children/adolescents, young adults and their parents prepare for an amputation.
Yes_______ No _______

If no, why not?
INSERT OPENING SCRIPT HERE

1. How old were you at time of amputation? ____ years old
2. How old are you now? _____ years old
3. What do you remember being told about why you were to have an amputation?
   ____
4. Did you feel like you were involved in deciding whether to have the amputation?
   - ☐ Yes (Please explain what you remember about the decision-making process):
     ____
   - ☐ No (Please explain: ____)
   - ☐ Don't remember
5. To the best of your memory, how long was it between the time you were told you were getting an amputation and when it actually happened?
   ____

6. Who told you that you needed to have an amputation? (Check all that apply)
   - ☐ Parent/Caregiver
   - ☐ Doctor
   - ☐ Nurse
   - ☐ Counselor
   - ☐ Don't remember
   - ☐ Other: ______________________________

7. Was the actual surgery described?
   - ☐ Yes, Please explain: ______________________________
   ____
   - ☐ No, Please explain: ______________________________
   - ☐ Don't remember

8. Were you told how you would feel after the surgery?
   - ☐ Yes, Please explain: ______________________________
   ____
   - ☐ No, Please explain: ______________________________
   - ☐ Don't remember

9. Were you given information about the amputation and/or alternative options?
   - ☐ Yes, Please explain: ______________________________
   ____
   - ☐ No, Please explain: ______________________________
   - ☐ Don't remember

10. Were you told how you would feel after?
    - ☐ Yes, Please explain: ______________________________
    ____
    - ☐ No, Please explain: ______________________________
    - ☐ Don't remember

11. How was the information given to you? Check all that apply.
    - ☐ Pamphlets/printed materials
    - ☐ Web resources
    - ☐ Verbally by health care provider
    - ☐ Verbally by parent
    - ☐ Other: ______________________________

12. Did you feel like you were given appropriate/enough information regarding the amputation?
    - ☐ Yes, Please explain: ______________________________
    ____

- ☐ No, Please explain:____
- ☐ Don't remember

13. What about alternative options?
    - ☐ Yes, Please explain: ____
    - ☐ No, Please explain:____
    - ☐ Don't remember

14. What feelings do you remember having surrounding the decision to proceed with amputation?

    ____

15. Do you think your parents felt like they had other options when it came to the decision to pursue amputation?
    - ☐ Yes, Please explain: ____
    - ☐ No, Please explain:____
    - ☐ Not sure, Please explain: ____________________________________

16. Who in your family do you think had the hardest time with you having to have an amputation?

    ____

17. Why do you think that was?

    ____

18. From what you know now, what do you feel would have been helpful for you to have told before the time of amputation?

    ____

19. At any point prior to your amputation, did you meet another person(s) who had undergone an amputation?
    - ☐ Yes
        1. Was this helpful?
            - ☐ Yes ____________________________________
            - ☐ Somewhat ________________________________
            - ☐ No ____________________________________
    - ☐ No
        2. Would you have liked to?
            - ☐ Yes ____________________________________
            - ☐ Not sure _________________________________
            - ☐ No ____________________________________

20. Please describe what you remember your life was like for the first few months following the amputation:

    ____

21. Did you feel you were prepared **emotionally** for the amputation?
    - ☐ Yes, Please explain what was helpful: ____________________
    - ☐ Not sure, Please explain: ____________________
    - ☐ No, Please explain:____________________

What would have been helpful? ____________________

22. Did you feel you were prepared **emotionally** for life **after** the amputation?
    - ☐ Yes, Please explain what was helpful: ____________________
    - ☐ Not sure, Please explain: ____________________
    - ☐ No, Please explain:____________________

What would have been helpful? ____________________

23. Did you feel you were prepared **physically** for life **after** amputation?
    - ☐ Yes, Please explain what was helpful: ____________________
    - ☐ Not sure, Please explain: ____________________
    - ☐ No, Please explain:____________________

What would have been helpful? ____________________

24. What would you say were the three hardest parts of the amputation process?
    1. ____________________
    2. ____________________
    3. ____________________

25. What would you recommend that health care providers do to help children/adolescents/young adults prepare for an amputation? (If prompts are needed, ask about emotionally, teaching materials, meetings others, time to get ready?)____________________

26. How do you feel health care providers should help prepare parents for their child’s amputation? (If prompts are needed, ask about emotionally, teaching materials, meetings others, time to get ready?)
    ____

27. Was there anything that you remember about the care that you received prior to the surgery that made you angry?
    - ☐ Yes, Please explain: ____________________
    - ☐ No, Please explain:____________________
    - ☐ Don’t remember

28. Was there anything that anyone said to you prior or immediately after the surgery that made you angry?
    - ☐ Yes, Please explain: ____________________
    - ☐ No, Please explain:____________________

☐ Don’t remember

29. Anything that the care team did or said that you remember helped you feel more prepared or comfortable?
    - ☐ Yes, Please explain: ____
    - ☐ No, Please explain:____
    - ☐ Don’t remember

30. Is there anything that we missed that you would like to tell us about regarding helping a child, adolescent or young adult get ready or be prepared for an amputation?

    ____

Supplemental Table S2: Current Resources for Children and Adolescents with Osteosarcoma

Resources on Make It Better (MIB) Website.

Resource	Resource Definition	Resource Format (In Person or Online)
MIB Ambassador Agents https://www.mibagents.org/mib-programs	The MIB Ambassador Agents program connects osteosarcoma patients and families to osteosarcoma survivors and their families. Ambassador Agents are individuals who have previously been diagnosed with osteosarcoma or who are connected to a loved one with osteosarcoma.	In Person and Online
MIB Prayer Agents	Prayer Agents is a community of families who have been deeply affected by osteosarcoma. MIB Prayer Agents work to honor, pray, and remember those who have passed away.	In Person and Online
MIB Healing Hearts	Healing Hearts is MIB's bereavement program of group sessions and retreats and retreats for parents and siblings. It is led by a life coach that specializes in End of Life and Grief coaching and works to provide education on experiencing grief and what to expect, as well as coping materials and support.	In Person
MIB Smart Patients https://www.smartpatients.com/communities	Smart Patients is an online community for patients 18 years and older and caregivers impacted by a variety of chronic diseases, illnesses, and they specifically have a group for osteosarcoma. This works with MIB Agents to create a network for families to share support and information with one another.	Online
Share Your Story	This platform on MIB Agents website allows survivors, siblings, and parents to share their stories about their experiences with osteosarcoma.	Online
Factor Conference	MIB Agents has an annual conference called FACTOR (Funding, Awareness, Collaboration, Trials, Osteosarcoma, and Research) and is designed to bring together the leading researchers, clinicians, and surgeons, along with families, survivors, patients, and siblings.	In Person

Resources on the Osteosarcoma Institute Website

Resource	Resource Definition	Resource Format (Online vs. In Person)
OSI Connect (Online) - https://osinst.org/osi-connect/	This is a free resource through the Osteosarcoma Institute for patients who have recently been diagnosed with osteosarcoma - it is designed to help answer a variety of questions at the pace and in regard to the questions that a patient or their family asks. This resource helps patients find answers to their questions from an osteosarcoma physician. It is also offered in Spanish.	Online
The Osteosarcoma Collaborative - https://oscollaborative.org/	This is an organization that provides those who have been diagnosed with osteosarcoma with a variety of resources such as osteosarcoma decision aid, patient stories, financial resources, and resources for bereaved families.	Online
Stupid Cancer - https://stupidcancer.org/	This organization supports adolescents and young adults with cancer through providing online, age-appropriate resources that help to navigate a variety of differing parts of life, including mental health, fertility, sex, wellness, caregiving, adulting, advocacy, and survivorship.	Online

In-Person Camp Resources

Resource	Resource Definition	Resource Format
Osteosarcoma Institute - Happiness Is Camping - https://www.happinessiscamping.org/	This program provides camps for patients with cancer between the ages of 6 and 15, as well as their families. They have camps for a wide variety of differing cancers (osteosarcoma camp, sickle cell camp, etc.).	In Person
MIB Summer Camp Directory (United States) - https://www.alexslemonade.org/childhood-cancer/for-families/summer-camps	There are a variety of camps offered that MIB has organized for each state that are specifically for children who have been diagnosed with various types of cancer.	In Person and Online
